# Adenoviral Transduction of Human Acid Sphingomyelinase into Neo-Angiogenic Endothelium Radiosensitizes Tumor Cure

**DOI:** 10.1371/journal.pone.0069025

**Published:** 2013-08-02

**Authors:** Branka Stancevic, Nira Varda-Bloom, Jin Cheng, John D. Fuller, Jimmy A. Rotolo, Mónica García-Barros, Regina Feldman, Shyam Rao, Ralph R. Weichselbaum, Dror Harats, Adriana Haimovitz-Friedman, Zvi Fuks, Michel Sadelain, Richard Kolesnick

**Affiliations:** 1 Laboratory of Signal Transduction, Memorial Sloan-Kettering Cancer Center, New York, New York, United States of America; 2 Department of Radiation Oncology, Memorial Sloan-Kettering Cancer Center, New York, New York, United States of America; 3 Center for Cell Engineering, Memorial Sloan-Kettering Cancer Center, New York, New York, United States of America; 4 Department of Radiation and Cellular Oncology, University of Chicago, and the Ludwig Center for Metastasis Research, Chicago, Illinois, United States of America; 5 Vascular Biogenics Ltd., Or Yehuda, Israel; Institut Gustave Roussy, France

## Abstract

These studies define a new mechanism-based approach to radiosensitize tumor cure by single dose radiotherapy (SDRT). Published evidence indicates that SDRT induces acute microvascular endothelial apoptosis initiated via acid sphingomyelinase (ASMase) translocation to the external plasma membrane. Ensuing microvascular damage regulates radiation lethality of tumor stem cell clonogens to effect tumor cure. Based on this biology, we engineered an ASMase-producing vector consisting of a modified pre-proendothelin-1 promoter, *PPE1(3x)*, and a hypoxia-inducible dual-binding *HIF-2α-Ets-1* enhancer element upstream of the *asmase* gene, inserted into a replication-deficient adenovirus yielding the vector *Ad5H2E-PPE1(3x)-ASMase*. This vector confers ASMase over-expression in cycling angiogenic endothelium *in vitro* and within tumors *in vivo*, with no detectable enhancement in endothelium of normal tissues that exhibit a minute fraction of cycling cells or in non-endothelial tumor or normal tissue cells. Intravenous pretreatment with *Ad5H2E-PPE1(3x)-ASMase* markedly increases SDRT cure of inherently radiosensitive MCA/129 fibrosarcomas, and converts radiation-incurable B16 melanomas into biopsy-proven tumor cures. In contrast, *Ad5H2E-PPE1(3x)-ASMase* treatment did not impact radiation damage to small intestinal crypts as non-dividing small intestinal microvessels did not overexpress ASMase and were not radiosensitized. We posit that combination of genetic up-regulation of tumor microvascular ASMase and SDRT provides therapeutic options for currently radiation-incurable human tumors.

## Introduction

Recent investigations using high single dose radiotherapy (SDRT) suggest that it acts by a biologic mechanism distinct from the mechanism operative in classical fractionated radiotherapy. The fractionated approach is based on the notion that normal cells repair ionizing radiation (IR)-induced double strand breaks (DSBs) more efficiently than tumor cells, which display dysregulated repair. Hence at the low dose range used in each treatment session (1.8–2.0 Gy), fractionation progressively protects normal tissue relative to tumor, enabling buildup of tumor dose as fraction number increases [1], [2]. Treatment exposures are repeated daily (5/wk) until maximal tolerable normal tissue doses are reached. Thus the tumor dose delivered is frequently determined by normal tissue toxicity rather than by dose required for tumor cure. The overall local cure with the fractionated approach is ∼65% of all tumors treated with curative intent [3], with a well-defined rank ordering of tumor curability based on tumor type [4]. Over the past decade, implementation of intensity modulation (IMRT) and image guidance (IGRT), which improve precision in tumor targeting, have reduced normal tissue exposure and enabled alternative therapeutic strategies, such as high SDRT. Early clinical experience with SDRT shows ∼90% local tumor cure in most tumors at a dose of 24 Gy, irrespective of tumor type [5]–[8], including tumors resistant to fractionated schemes. This SDRT dose level is considered far too low for cure, as predicted by classic radiobiologic LQ model formalism [9], [10]. The high SDRT cure rate and the lack of rank ordering of cure by tumor type have raised the question whether the two radiation methods are distinct mechanistically. Despite use of advanced tumor targeting technology large cohorts of patients remain ineligible for this highly-curative therapy because of risk of toxicity, as their tumors either adhere to or engulf critical normal structures, inseparable for tumor-selective SDRT delivery.

Our laboratory [11]–[14] demonstrated that tumor stem cell clonogen (SCC) lethality after SDRT, within the clinically-relevant range of 8–25 Gy, is conditionally-linked to an early wave (0.5–6 h) of acid sphingomyelinase (ASMase)**-**induced apoptosis in the microvascular endothelium of exposed tissue, and that the coupling of these events mediates SDRT tumor cure. This model is supported by studies in which tumors grown in *asmase^−/−^* mice, which provide apoptosis-resistant vasculature, are refractory to SDRT tumor cure. Mechanistically, high SDRT induces ASMase trafficking to endothelial plasma membrane within seconds to minutes of irradiation, generating ceramide therein [13], an event obligate for endothelial apoptosis [15]. This membranous event appears independent of DNA damage repair as tumors in SCID mice, defective in DNA-PKcs involved in DSB repair, are equally sensitive to SDRT-induced endothelial apoptosis and tumor cure as wild type littermates [14]. Endothelial vulnerability to IR-induced damage appears related to a 20-fold higher ASMase expression in endothelium than in any other mammalian cell investigated, and to preferential expression of a specialized secretory ASMase (S-ASMase) form in endothelium [16]. Due to its unique biophysical properties, the generated ceramide reorganizes the plasma membrane, forming signaling domains therein termed ceramide-rich platforms (CRPs). These macrodomains serve as sites for protein oligomerization and transmembrane signaling [17] of apoptosis, and are absent in cells lacking ASMase. Although the exact sequence of events that couple ASMase-mediated endothelial apoptosis to tumor cure remains under investigation, our preliminary data indicate that microvascular dysfunction signals impairment of homology driven-repair of potentially-lethal DNA DSBs in tumor SCCs ([11], [14], and Thin, Kolesnick and Fuks, unpublished).

Here we explore the hypothesis that amplification of ASMase-induced ceramide generation would enhance platform formation, microvascular dysfunction, and tumor response following SDRT. To investigate this concept, we engineered a replication-defective adenoviral serotype 5 vector to contain a modified pre-proendothelin-1 promoter *PPE1(3x)*, which confers specificity to proliferating endothelial cells, upstream of the ASMase gene, yielding a construct termed *Ad5H2E-PPE1(3x)-ASMase*. In vivo, systemic treatment with *Ad5H2E-PPE1(3x)-ASMase* confers ASMase over-expression exclusively in angiogenic endothelium within tumors, enhancing endothelial sensitivity to radiation-induced apoptosis and tumor cure, affording conversion of radiation-incurable tumors into permanent tumor ablation. We propose such selective tumor versus normal tissue radiosensitization might provide a solution for the current restriction of SDRT usage in tumors encroaching on critical normal organs.

## Results

### IR induces CRP generation in mouse tumor endothelial cells

**Figure 1:**
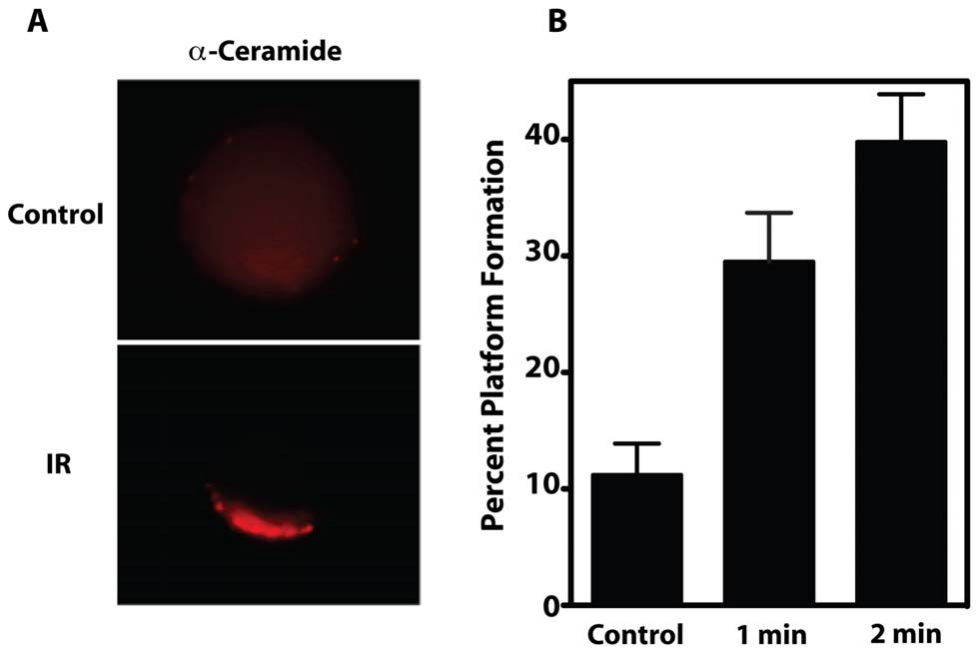
IR induces CRP formation on the surface of purified tumor endothelium. (**A**) Representative image of the clustering of ceramide into platforms on the outer leaflet of the plasma membrane of purified tumor endothelial cells at 1 min post 15 Gy. For these studies, irradiated cells were stained with Texas Red-labeled anti-ceramide antibody, and platforms identified as in Materials and Methods. Images are representative of over 30% of cells from 3 experiments in which over 100 cells were analyzed. (**B**) Time-dependent generation of platforms in tumor endothelial cells. CRPs in tumor endothelial cells irradiated *ex vivo* with 15 Gy were quantified as in Materials and Methods. Data (mean ± 95% CI) are collated from 3 experiments in which 200 cells were analyzed per point.

We recently showed that IR activates ASMase to generate ceramide, leading to formation of CRPs, defined as cell surface macrodomains of 500 nm up to several microns in size, within 30 sec of irradiation of cultured bovine aortic endothelial cells (BAEC) [18]. This event peaked at 1 min post-irradiation, was dose-dependent from 1–15 Gy and maximal at 11 Gy (ED_50_∼5 Gy). To confirm that CRPs form in tumor endothelium upon irradiation, endothelial cells were purified to homogeneity from MCA/129 fibrosarcomas implanted in the hind limb of sv129/BL6 mice by positive and negative selection as published [12], and irradiated *ex vivo*. Similar to BAEC, tumor endothelial cells isolated to homogeneity showed rapid ASMase activation (not shown) and formation of platforms enriched in ceramide (**Fig. 1**) in 40±4% of the total population at 2 min post 15 Gy (the 50% tumor control dose for MCA/129 fibrosarcomas), compared to 11±3% of unstimulated controls (*P*<0.05).

### Overexpression of ASMase via *Ad5H2E-PPE1(3x)-ASMase* leads to increased ASMase activity, BAEC radiosensitization and attenuation of bFGF protection

**Figure 2:**
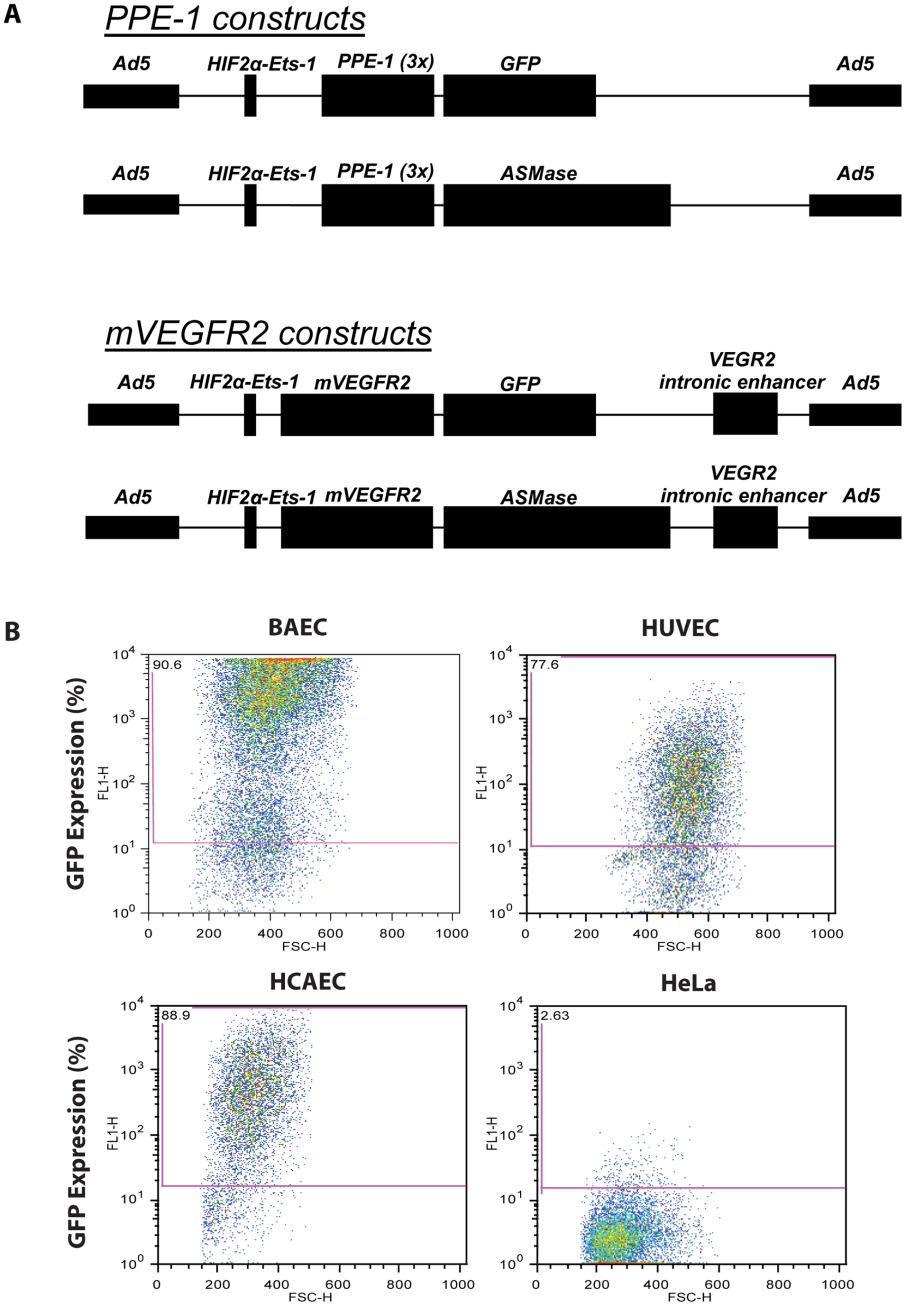
Infection with *Ad5H2E-PPE1(3x)-GFP* confers GFP expression specific to endothelial cells. (**A**) Schematic representation of gene therapy vectors used to overexpress the GFP reporter or human ASMase. (**B**) Primary cultures of bovine and human endothelial cells (BAEC, HUVEC and HCAEC) and non-endothelial cells (HeLa) were infected with *Ad5H2E-PPE1(3x)-GFP*. GFP expression was measured in live cells following detachment 72 h post-infection by flow cytometry.

We hypothesized that genetic amplification of ASMase activity might increase CRP formation in tumor endothelium, radiosensitizing tumors. To test this notion, we initially developed a set of replication-defective adenovector constructs to specifically increase human ASMase expression in angiogenic endothelium. Expression of the ASMase target gene was placed under the control of an endothelial specific promoter, either the murine *VEGFR2* (*Flk-1*), human *VEGFR2* (*KDR*) or murine *PPE* promoter, and enhanced with the hypoxia-inducible dual binding element *HIF-2α-Ets-1* from the murine *VEGFR2* promoter (for details on construct design see Materials and Methods, and Supporting Materials and Methods in File S1) [19]. A complementary set of constructs was engineered to express GFP or GFP-Luciferase utilized as reporters for optimization purposes. Enhancer-promoter activity of individual constructs was initially evaluated in cultured endothelium by assessing Luciferase or GFP expression intensity. Empirically, we determined that ASMase expression driven by HIF-2α-Ets-1 [19] upstream of the endothelial-specific modified *PPE1(3x)* promoter [20], [21] was most suitable for our experiments (see Supporting Results in File S1), and subsequently engineered that construct into an adenovirus serotype 5 (E1/E3 deleted) **(** **Fig.** **2A** **)**.

The specificity of the generated virus for endothelial cells was tested *in vitro*. We observed target gene expression following transduction with *Ad5H2E-PPE1(3x)-GFP* that was specific for endothelium, as 91%, 78% and 89% of BAEC, Human Umbilical Vein Endothelial Cells (HUVEC) and Human Coronary Artery Endothelial Cells (HCAEC), respectively, were GFP-positive by flow cytometry **(** **Fig.** **2B** **)**. In contrast, minimal expression was detected in cells of non-endothelial origin, as only 3% of HeLa cells **(** **Fig.** **2B** **)** and 2% of Jurkat cells (data not shown) displayed GFP-positivity. Additionally, no significant adenovirus-induced toxicity was observed (data not shown).

**Figure 3:**
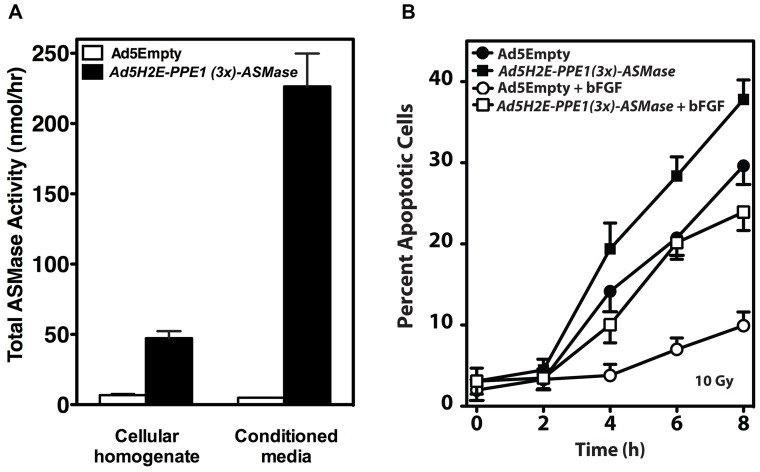
Overexpression of human ASMase via *Ad5H2E-PPE1(3x)-ASMase* increases lysosomal and secretory ASMase activity, radiosensitizes BAEC, and attenuates bFGF protection against radiation-induced apoptosis. (A) Cellular homogenates and serum-free conditioned media were harvested from BAEC transduced with *Ad5Empty* or *Ad5H2E-PPE1(3x)-ASMase* and assayed for ASMase activity using [^14^C-methylcholine]sphingomyelin as substrate in the presence of 1 mM EDTA (cellular homogenates) or 0.1 mM Zn^2+^ (conditioned media). (B) BAEC transduced with *Ad5Empty* or *Ad5H2E-PPE1(3x)-ASMase* were pre-treated with 1 ng/ml bFGF 15 minutes prior to 10 Gy irradiation. Apoptosis was assessed at various time points after IR by morphologic analysis following *bis*-benzimide staining. Data (mean ± SEM) are collated from 3 experiments performed in triplicate in which 400 nuclei were analyzed per sample.

*Ad5H2E-PPE1(3x)-ASMase*-mediated ASMase overexpression led to generation of physiologically-active enzyme, as ASMase activity increased 8.3-fold in homogenates of transduced BAEC from a baseline of 6.7±1.1 to 47.3±5.0 nmol/h (*P*<0.001) at 72 h post-infection **(** **Fig.** **3A** **)**. Concomitantly, a 46.1-fold increase in secreted ASMase activity was detected in conditioned media of *Ad5H2E-PPE1(3x)-ASMase* infected BAEC after 72 h from a baseline of 4.9±0.3 to 226.4±23.3 nmol/h (*P*<0.001). Thus 84.5% of the total synthesized ASMase activity is secreted upon *Ad5H2E-PPE1(3x)-ASMase* infection. Similar extent of secretion was achieved by others in B16-F10 cells transduced to overexpress ASMase using a constitutive CMV promoter in a retroviral vector [22]. That the form of ASMase found in conditioned media of *Ad5H2E-PPE1(3x)-ASMase-*transduced BAEC represents the S-ASMase alternatively processed form of ASMase first reported by Tabas and co-workers [23] was confirmed by the requirement for Zn+ addition to the assay buffer to detect enzymatic activity **(Fig. S1)**. These results suggest that similar to *Ad5H2E-PPE1(3x)-GFP, Ad5H2E-PPE1(3x)-ASMase* efficiently infects BAEC delivering the human ASMase gene properly expressed and processed by the cellular machinery to generate enzymatically-active protein.

ASMase overexpression increased radiation-stimulated CRP formation by 35-55% at 1–5 min following 10 Gy compared to *Ad5Empty* control (*P*<0.05 each), and conferred ∼40% increase in apoptosis at 8 h at all doses from 5–15 Gy (*P*<0.05 each, data shown for 10 Gy in **Fig.** **3B**), yielding a dose modifying factor of 1.35. Critically, ASMase overexpression attenuated the well-documented radioprotection elicited by the endothelial survival factor, basic fibroblast growth factor (bFGF), mimicking conditions expected in hypoxic tumor fractions. As shown in **Fig.** **3B**, bFGF confers radioprotection to *Ad5Empty*-transduced BAEC, leading to 87% reduction in apoptosis at 4–8 h after 10 Gy (*P*<0.05 for each time). Overexpression of ASMase via *Ad5H2E-PPE1(3x)-ASMase* antagonized the bFGF protective effect in BAEC. In fact, apoptosis levels after bFGF treatment in infected and irradiated cells were comparable to those observed in irradiated *Ad5Empty*-infected cells without bFGF treatment. In sum, ASMase overexpression increased radiation sensitivity of BAEC and overcame growth factor-conferred radioresistance. These findings may have application *in vivo* as tumors confer radioresistance in part by expression of angiogenic growth factors such as bFGF and VEGF, induced by hypoxia and HIF-1, that render protection to tumor endothelium [24].

### Ad5H2E-PPE1(3×) restricts target gene expression to angiogenic endothelium

**Figure 4:**
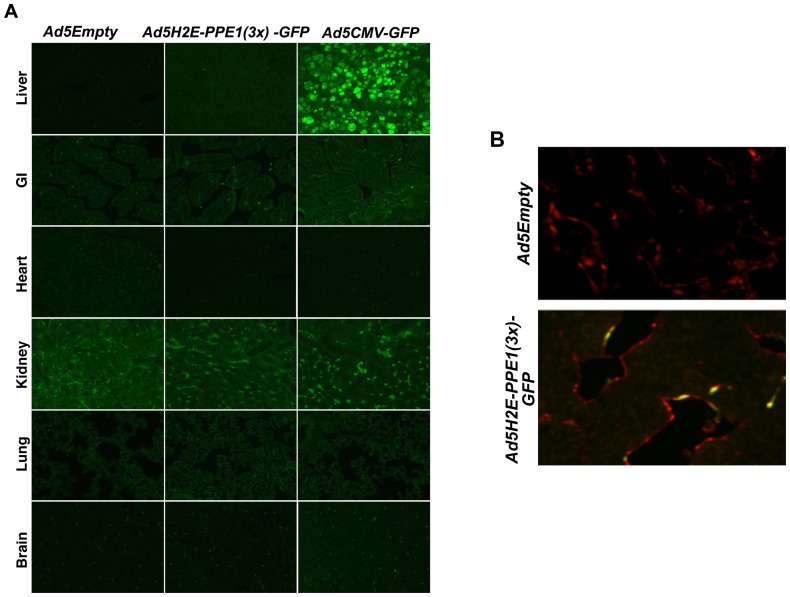
Intravenous administration of *Ad5H2E-PPE1(3x)-GFP* results in GFP expression selectively in tumor endothelium. 1×10^10^ PFU of *Ad5Empty*, *Ad5H2E-PPE1(3x)-GFP* or *Ad5CMV-GFP* were intravenously administered to MCA/129 fibrosarcoma-bearing sv129/BL6 mice. Five days post viral administration, normal tissues (**A**) and tumor tissue (**B**) were excised and GFP expression was visualized by standard fluorescence microscopy following staining with anti-GFP (green; **A** and **B**) and anti-Meca-32 (red; **B**) antibodies, as described in Materials and Methods. Shown are representative 200× images of 20 fields analyzed per sample. Note background autofluorescence in the kidney specimens.

Varda-Bloom et al. previously showed that the *PPE1(3x)* promoter directs gene expression specifically to the tumor vascular bed, yielding 35-fold higher expression in tumor vessels compared to the normal pulmonary vascular bed due to activity of an unknown transcriptional complex found exclusively in proliferating endothelium [20], [21]. Our studies corroborate these findings as intravenous administration of 1×10^10^ plaque-forming units (PFU) of *Ad5H2E-PPE1(3x)-GFP* to B16 melanoma-bearing C57BL6 and MCA/129 fibrosarcoma-bearing sv129/BL6 mice revealed specificity of GFP reporter expression in angiogenic endothelium. Co-immunostaining of tumor cross-sections **(** **Fig. 4** **)** with an antibody to the endothelial selective cell surface marker MECA-32 (red) and an antibody to GFP (green) revealed GFP positive endothelial cells (typical yellow co-staining endothelium depicted in **Fig. 4B**) as early as two days following administration of 1×10^10^ PFU of *Ad5H2E-PPE1(3x)-GFP* (**Fig. S2A**). Peak reporter gene expression was detected 5 days post administration in 5.4%±0.9 and 5.9%±0.5 of endothelial cells within MCA/129 fibrosarcoma and B16 melanoma tumor models, respectively (**Fig. S2B**), which persisted for an additional 9 days (*P*>0.005; not shown). Equivalent expression was observed in tumors of varying size from 64–203 mm^3^ (**Fig. S2C**). In contrast, no detectable GFP expression was observed following intravenous administration of 1×10^10^ PFU of *Ad5H2E-PPE1(3x)-GFP* in endothelium (or epithelium) of liver, GI tract, heart, kidney, lung, brain (**Fig. 4A**), spleen, skin or pancreas (data not shown). However, high levels of green fluorescence were detected in hepatocytes following intravenous administration of the tissue non-specific positive control, *Ad5CMV-GFP*, in which GFP expression is driven by the constitutive *CMV* promoter. These data are consistent with data of Harats and co-workers showing that the *PPE1(3x)* promoter confers significantly higher expression in proliferating endothelium than the *CMV* promoter [20], [21]. These data indicate that the *Ad5H2E-PPE1(3x)* construct nearly exclusively targets neo-angiogenic endothelium in tumor-bearing mice.

### Restoration of ASMase in tumor endothelium of *asmase^−/−^* mice restores sensitivity of MCA/129 fibrosarcomas to radiation

**Figure 5:**
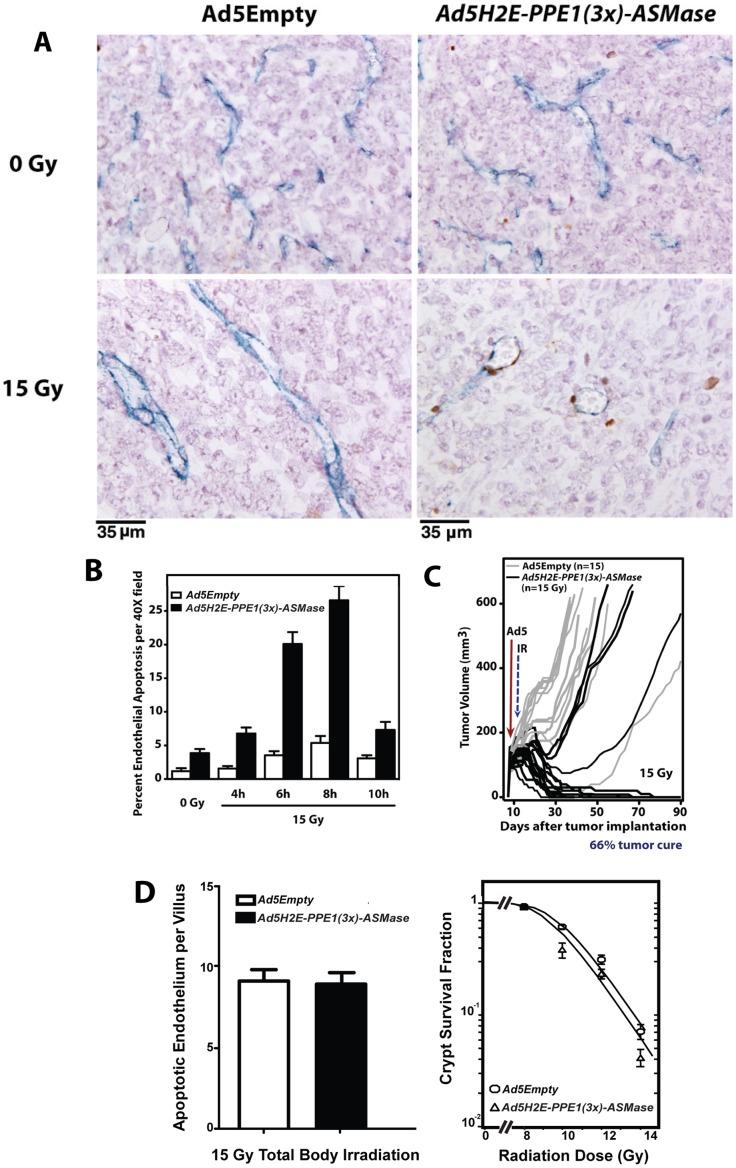
Expression of ASMase in endothelium of MCA/129 fibrosarcomas implanted in *asmase^−/−^* mice restores radiation-induced endothelial apoptosis and tumor cure. MCA/129 fibrosarcoma cells (10^6^, resuspended in PBS) were injected intra-dermally into the right hind limb of *asmase^−/−^* mice (A, B, C) and tumor volume (based on caliper measurements) was calculated daily according to the formula by Kim et al. [54]. At 90–130 mm^3^, 1×10^10^ PFU of *Ad5Empty* or *Ad5H2E-PPE1(3x)-ASMase* was administered intravenously. 5 days post viral administration tumors were locally irradiated with 15 Gy or left untreated. (A) Representative cross sections of MCA/129 fibrosarcoma excised from unirradiated animals (upper panel) and at 6 hours post 15 Gy (lower panel), and co-stained for an endothelial-specific marker (Meca-32, blue) and apoptosis (TUNEL; brown). (B) Quantitation of the effect of *Ad5H2E-PPE1(3x)-ASMase* treatment on radiation-induced endothelial cell apoptosis. Data (mean ± SEM) represent TUNEL-positive endothelial cells quantified from 20 fields/tumor (400× magnification) and 2 tumors per group. (C) Impact of treatment with *Ad5Empty* (gray lines) or *Ad5H2E-PPE1(3x)-ASMase* (black lines) followed by 15 Gy on MCA/129 fibrosarcoma response. N equals number of animals per group. Tumors were measured daily up to 40 days and twice weekly thereafter. (D) Lack of impact of *Ad5H2E-PPE1(3x)-ASMase* on small intestinal radiation sensitivity. Mice, pre-treated for 5 days with *Ad5Empty* or *Ad5H2E-PPE1(3x)-ASMase,* were subjected to 15 Gy (left) or 8–14 Gy (right) total body irradiation known to cause GI damage. Mice were sacrificed at 4 h (left) for endothelial apoptosis measurement or after 3.5 days (right) for Crypt Microcolony Assay as standardized in [18], [27]. Endothelial apoptosis was identified by microscopic co-detection of TUNEL (brown) and MECA-32 (blue) staining. Data (mean ± SEM) were compiled from 2 mice each, analyzing apoptotic cells in the lamina propria of 20 intact crypt-villus units per mouse. Crypt survival curves were calculated using 2 mice per dose analyzing 5–10 crypt-villus units per mouse by least square regression analysis, with a modification of the FIT software program [55]. The program fits curves by iteratively weighted least squares to each set of dose–survival data, estimates covariates of survival curve parameters and corresponding confidence regions, and plots the survival curve.

Previous studies from our laboratory indicated that SDRT-induced apoptotic damage to the endothelial compartment, mediated by ASMase, is necessary for SDRT-induced tumor cure [12]. Here we show by analysis of tumor cross-sections doubly-immunostained with antibody to the endothelial selective cell surface marker MECA-32 (blue in **Fig. 5A**) and TUNEL for apoptosis (brown in **Fig. 5A**) that restoration of ASMase expression via *Ad5H2E-PPE1(3x)-ASMase* in endothelium of tumors transplanted in *asmase^−/−^* mice restores IR-induced apoptosis. While locally-irradiated MCA/129 fibrosarcomas implanted in *asmase^−/−^* mice did not display significant endothelial apoptosis at 4–10 h after 15 Gy (**Fig. 5B**; *P*>0.05 at all times vs. control), restitution of ASMase expression with *Ad5H2E-PPE1(3x)-ASMase* restored endothelial apoptosis to 27±2% at 8 h respectively post 15 Gy (*P*<0.005 vs. unirradiated control), without evidence of tumor cell apoptosis (<2%).

Moreover, *Ad5H2E-PPE1(3x)-ASMase*-mediated restoration of ASMase expression in tumor endothelium of *asmase^−/−^* mice restored MCA/129 fibrosarcoma radiosensitivity. We normally achieve 45–50% local cure of MCA/129 fibrosarcoma implanted in *asmase^+/+^* mice after 15 Gy, reduced to <5% in *asmase^−/−^* littermates [12]. The current studies recapitulate our previous findings demonstrating local tumor cure, confirmed at autopsy, in only 6.6% of *asmase^−/−^* mice with 15 Gy (n = 15; **Fig. 5C** shown as grey lines), not different than the spontaneous cure rate. Selective ASMase re-expression by *Ad5H2E-PPE1(3x)-ASMase* in tumor microvasculature of *asmase^−^* ^/*−*^ mice restored the IR wild type response to 66% tumor cure (10 out of 15 mice shown as black lines measured daily). Collectively, these data show that restitution of ASMase expression specifically in tumor endothelium restores endothelial sensitivity to IR-induced apoptosis, re-engaging the vascular component of tumor response to IR, conferring cure. It is our working hypothesis that these events occur despite low-level tumor endothelial transduction because almost all ASMase synthesized by endothelium is secreted (>80% as shown in **Fig. 3A**). Furthermore, these studies provide genetic proof-of-concept that endothelial cell apoptosis couples high SDRT to tumor response, as *Ad5H2E-PPE1(3x)-ASMase* restored neo-angiogenic ASMase expression but not ASMase expression in any other cell of the *asmase^−/−^* mouse.

### Ad5H2E-PPE1(3×) does not sensitize normal tissue to SDRT

The above studies using *Ad5H2E-PPE1(3x)-GFP* demonstrate target gene expression specific for angiogenic endothelium, with no detectable expression in normal tissue endothelium (**Fig. 4A**). Nonetheless, our published data show direct damage to normal tissue endothelium mediates SDRT organ damage in lung, brain and the GI tract [15], [25], [26]. To address whether targeting tissue endothelium, as evidenced by our *Ad5H2E-PPE1(3x)-GFP* reporter studies, determines radiation outcome, we explored impact of systemic *Ad5H2E-PPE1(3x)-ASMase* administration on radiation damage to normal intestinal mucosa. A widely-utilized robust assay exists, the Clonogenic Assay of Withers and Elkind [27] for monitoring the extent of damage to small intestinal crypts. In fact, we used this assay in our prior genetic studies that linked intestinal microvascular damage to crypt and GI lethality in experimental mice [25], [28]. Pre-treatment with *Ad5H2E-PPE1(3x)-ASMase* did not impact endothelial apoptosis in wild-type mice exposed to the 100% lethal dose of 15 Gy total body irradiation (Fig. 5D, left – note baseline endothelial apoptosis is undetectable and in the absence of vector 15 Gy yields 9.2±0.5 apoptotic endothelium per villus [18]). Furthermore, *Ad5H2E-PPE1(3x)-ASMase* pre-treatment did not alter dose-dependent crypt survival after 8–14 Gy (**Fig. 5D**, right), representing the entire spectrum of doses known to injure murine GI mucosa (dose-modifying factor of 1.05±0.29). These studies indicate specificity of our *Ad5H2E-PPE1(3x)-ASMase* gene therapy to radiosensitize dividing endothelium of tumors, a pre-requisite for clinical applicability.

### Overexpression of ASMase in tumor endothelium radiosensitizes MCA/129 fibrosarcomas and B16-F1 melanomas

**Figure 6:**
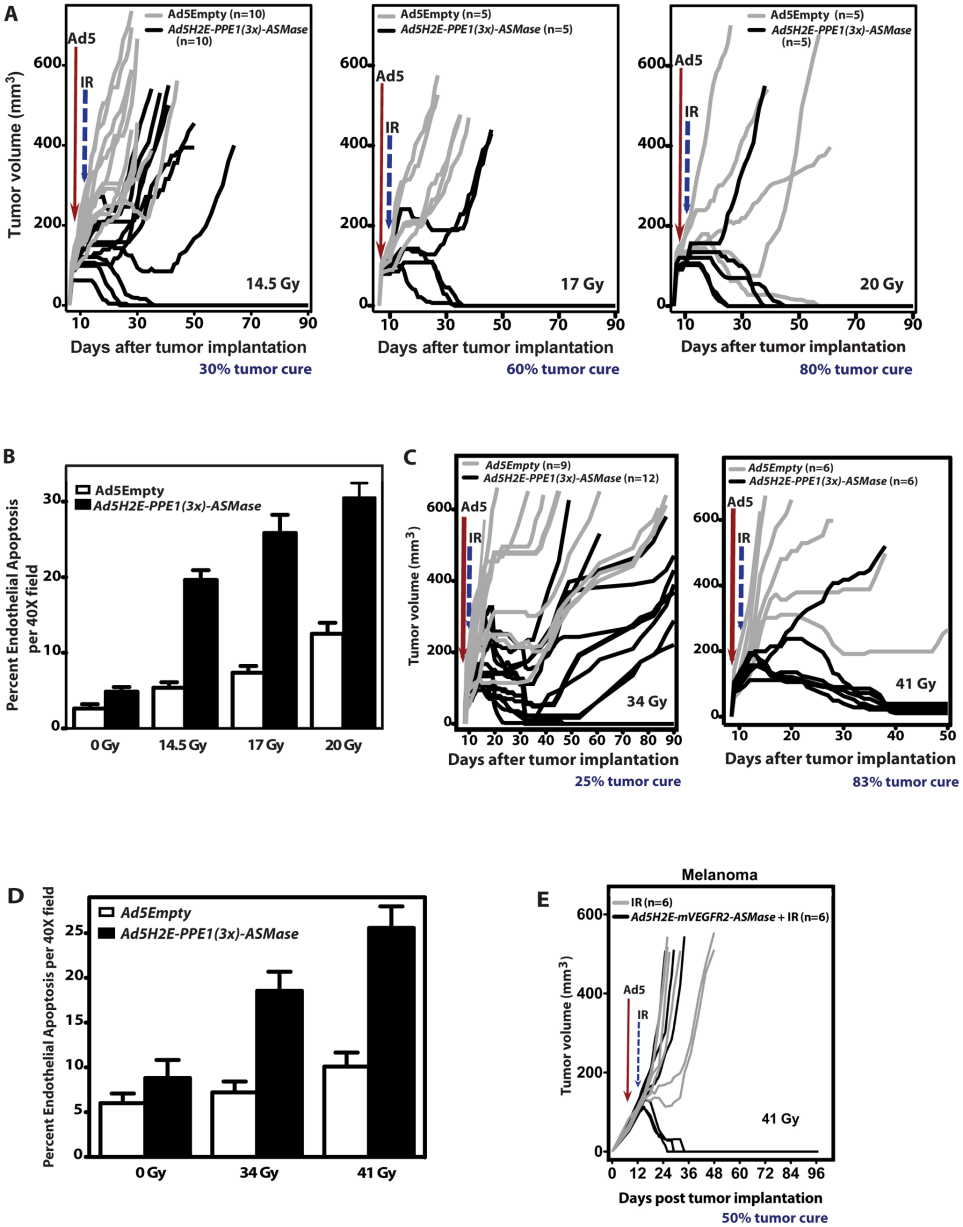
ASMase overexpression in tumor endothelium radiosensitizes MCA/129 fibrosarcomas and B16 melanomas. 1×10^10^ PFU of *Ad5Empty* or *Ad5H2E-PPE1(3x)-ASMase* was administered intravenously to MCA/129 fibrosarcoma- (**A,B**) and B16 melanoma (**C,D**)-bearing sv129/BL6^JAX^ mice, as in Figure 5. Five (**A**,**B**) or four (**C,D**) days post virus administration tumors were locally irradiated with 14.5, 17 Gy and 20 Gy (**A,B**), or 34 and 41 Gy (**C,D). (A,C**) Response of MCA/129 fibrosarcoma **(A)** and B16-F1 melanoma **(C)** to *Ad5Empty* (gray lines) or *Ad5H2E-PPE1(3x)-ASMase* (black lines) followed by IR. N = animals/group. Tumors were measured daily up to 40 days and twice weekly thereafter. Tumor cure was confirmed by local biopsy. (**B**,**D**) Quantitation of the impact of *Ad5H2E-PPE1(3x)-ASMase* on radiation-induced endothelial cell apoptosis within MCA/129 fibrosarcomas **(B)** and B16-F1 melanomas **(D)** implanted into sv129/BL6^JAX^ *asmase^+/+^* mice. Data (mean ± SEM) represent TUNEL-positive endothelial cells quantified from 20 (400× magnification) fields/tumor using 2 tumors/group. (**E**) ASMase overexpression in tumor endothelium using the murine *VEGFR2* promoter radiosensitizes B16 melanoma. 2×10^10^ PFU of *Ad5H2E-mVEGFR2-ASMase* was administered intravenously to B16 melanoma-bearing sv129/BL6^JAX^ mice. Five days thereafter tumors were locally irradiated with 41 Gy. B16 melanoma response to *Ad5H2E-mVEGFR2-ASMase* and irradiation (black lines) or radiation alone (gray lines) are presented as tumor volumes.

Whereas *in vitro* studies showed that ASMase overexpression in BAEC leads to apoptosis radiosensitization, we tested whether genetic ASMase up-regulation leads to radiosensitization of wild-type vasculature *in vivo,* improving tumor response to SDRT. For these studies we used a commercially-available sv129/BL6 mouse strain from Jackson Laboratories, which we term sv129/BL6^JAX^, as host. This strain displays significantly greater resistance to endothelial cell apoptosis than our in house sv129/BL6^SKI^ strain by an unknown mechanism, and in our experience right-shifts tumor responses. Hence the 50% tumor control dose (TCD50) for fibrosarcomas increases from ∼15 Gy in sv129/BL6^SKI^ hosts to ∼30 Gy in sv129/BL6^JAX^ hosts, while we do not cure melanomas in either background. However, the sv129/BL6^JAX^ strain has strict batch-to-batch quality control as these commercial mice are the product of heterozygous mating of pure sv129 and C57BL6 mouse strains, while our propagated colony is interbred as an sv129/BL6 strain and is thus subject to genetic drift. A full tumor control profile of 100 mice defined a TCD50 at 90 days of 29.8±0.9 Gy in sv129/BL6^JAX^ mice (data not shown). Consistent with these data, **Fig. 6A** shows that local tumor exposure of *Ad5Empty*-treated sv129/BL6^JAX^ hosts bearing fibrosarcomas to 14.5 Gy and 17 Gy SDRT leads to no cures, while 20% cure was achieved with 20 Gy (gray lines). Concomitant with lack of MCA/129 fibrosarcoma tumor response in sv129/BL6^JAX^ mice to 14.5 Gy and 17 Gy, irradiated tumors displayed minimal increase in endothelial apoptosis at 6 h after IR (**Fig. 6B** **;** *P*>0.05 each vs. unirradiated control). However, genetic ASMase up-regulation in tumor microvasculature via *Ad5H2E-PPE1(3x)-ASMase* led to significant increase in endothelial apoptosis to 20±1%, 26±2% and 31±2% following 14.5 Gy, 17 Gy and 20 Gy, respectively (**Fig. 6B**; *P*<0.005 each), and to local cures in 30%, 60% and 80% of fibrosarcomas following these doses, respectively (**Fig. 6A** black lines; *P*<0.05 each vs. *Ad5Empty*-treated controls). Hence, *Ad5H2E-PPE1(3x)-ASMase* pre-treatment reduces the iso-effective dose for 20–30% tumor cure by approximately 5.5 Gy from 20 Gy to 14.5 Gy.

In contrast to MCA/129 fibrosarcoma, which are curable in our laboratory, B16 melanomas in sv129/BL6^JAX^ mice cannot be cured up to a dose of 41 Gy **(** **Fig. 6C, right panel** **)**, well beyond the range of SDRT doses in current clinical usage. Consistent with our hypothesis that extent of vascular injury determines outcome of SDRT, B16 melanomas display <10% endothelial cell apoptosis up to 41 Gy **(** **Fig. 6D** **)**. However, genetic ASMase overexpression in the angiogenic compartment of radiation-incurable B16-F1 melanomas resulted in significant increase in the radiation-induced apoptotic response (19±2% vs. 7±1% at 34 Gy, and 27±2% vs. 10±2% at 41 Gy; *P*<0.005 each; **Fig. 6D**). While *Ad5H2E-PPE1(3x)-ASMase* treatment had no significant effect on baseline tumor growth (*P*>0.1; not shown), it conferred local tumor cure in 3 of 12 animals (25%) following 34 Gy SDRT (**Fig. 6C, left panel**) and 5 out of 6 animals after 41 Gy (83%; **Fig. 6C, right panel**). Thus *Ad5H2E-PPE1(3x)-ASMase* reduces the threshold for tumor cure from above 41 Gy to ≤34 Gy, at least a 7 Gy reduction in curative dose. Overall these data show that genetic ASMase up-regulation with *Ad5H2E-PPE1(3x)-ASMase* has a large radiosensitizing effect on mouse tumors, left shifting the dose response for the curable fibrosarcoma, and curing radioresistant melanomas.

To provide additional evidence for the specificity of ASMase overexpression in radiosensitizing tumor neo-angiogenic vasculature, we repeated the entire body of work using a second promoter, the murine *VEGFR2* promoter. Like the *PPE* promoter, the murine *VEGFR2* promoter requires a transcriptional element found in dividing neo-angiogenic cells for optimal activity [29]. **Fig. 2A** shows our adenovector comprised of the same *HIF-2α-Ets-1* enhancer used in the construction of *Ad5H2E-PPE1(3x)-ASMase* upstream of the murine *VEGFR2* promoter. Additionally an intronic enhancer (IE) from the murine *VEGFR2* gene required for optimal promoter function [30] is included 3′ to the promoter. Like *Ad5H2E-PPE1(3x)-ASMase,* this *Ad5H2E-mVEGFR2-ASMase* construct was expressed at a high rate in endothelial cell lines in cultured BAEC, HCAEC and HUVEC (**Fig. S3**), but not in HeLa cells or Jurkat cells (not shown). In vivo, 5×10^10^ PFU of a *Ad5H2E-mVEGFR2-GFP* reporter did not express in the epithelial or endothelial compartment of any major organs including but not limited to liver, GI tract, heart, kidney, lung or brain (**Fig. S4**), but was found expressed in 5.1±1.8% of neo-angiogenic endothelium of MCA/129 fibrosarcoma. Expression was detected at day 3 after injection and was maximal at day 5. Furthermore, as with *Ad5H2E-PPE1(3x)-ASMase,* overexpression of ASMase in the neo-angiogenic vasculature of radiosensitive MCA/129 fibrosarcomas (**Fig. S5**) or radioincurable B16-F1 melanomas (**Fig. 6E**) resulted in marked radiosensitization of tumor cure. Direct comparison of the effectiveness of the two adenovectors shows, based on number of injected viral particles (vps), that *Ad5H2E-PPE1(3x)-ASMase* (1×10^10^ PFU injected/mouse  = 2.75×10^11^ vps) is 4-fold more potent than *Ad5H2E-mVEGFR-ASMase* (5×10^10^ PFU injected/mouse  = 1.1×10^12^ vps). These studies show that irrespective of the promoter used to specify tumor vasculature, ASMase overexpression in tumor vasculature radiosensitizes tumor cure.

## Discussion

The present studies reporting that genetic restoration of ASMase expression in neo-angiogenic microvessels of ASMase-null mice restores endothelial sensitivity to SDRT and tumor cure provide conclusive evidence that radiation-induced endothelial dysfunction is a fundamental component of the SDRT effect. Although *Ad5H2E-PPE1(3x)-GFP* or *Ad5H2E-mVEGFR2-GFP* conferred GFP reporter expression in only 5–6% of total tumor endothelium, the large anti-tumor effect following infection of such a small fraction of the tumor microvascular endothelium can be explained by the secretory nature of the *asmase* gene product, analogous to the bystander effect seen in many types of gene therapy [31]–[36]. While *Ad5H2E-PPE1(3x)-ASMase* infection yields both lysosomal and secretory forms of the enzyme, 50-fold more secreted enzyme was observed. Published data indicate secreted enzyme is endocytosed via the mannose 6-phosphate receptor, and re-enters the intracellular signaling compartment [23]. Hence, ASMase impact may not be restricted to transduced tumor endothelial cells capable of activating the *PPE1(3x)* promoter, i.e. those that are cycling (although this is not formally proven in the current study), as secreted enzyme would likely enhance radiosensitivity of neighboring tumor endothelium. Further, the ASMase effect observed in this work appears endothelial specific, as no significant increase in apoptosis following virus administration and irradiation was observed in tumor parenchymal cells. Nevertheless, published evidence suggests it may be possible to overexpress ASMase in some tumor parenchymal cells and achieve radiosensitization [22], a concept that requires additional investigation.

Another fundamental finding is the demonstration that extent to which SDRT tumor cure is enhanced depends on intensity of endothelial damage, monitored via the apoptotic response. Not only does *Ad5H2E-PPE1(3x)-ASMase* or *Ad5H2E-mVEGFR2-ASMase* treatment markedly left shift the dose response for cure of radiation sensitive MCA/129 fibrosarcomas, but confers cure onto radio-incurable B16 melanomas. As such, these data support development of an adenoviral gene therapy approach directed at targeting tumor vasculature with ASMase for SDRT potentiation. There are concerns that clinical use of adenoviral-based constructs in human tumor therapy may be limited by hepatic uptake or systemic antibody production against adenoviral capsid proteins, both potentially restricting bioavailabilty of injected construct. Concern also exists regarding safety of intravenous adenoviral gene therapy. Nonetheless, three recent Phase I-II trials reporting data using systemically-administered adenoviral constructs including one using the modified *PPE1(3x)* promoter we incorporated into *Ad5H2E-PPE1(3x)-ASMase* [37]–[39] indicate otherwise. These studies employed up to three repeated doses of 3×10^12^ vps without dose-limiting toxicity or maximally-tolerated dosing reached [37]–[39]. Furthermore, in one study, which employed a non-replicating adenoviral vector (Ad5, E1 deleted) consisting of modified *PPE1(3x)* promoter upstream of a fas-human TNF receptor chimera delivered via single bolus of 1×10^6^–3×10^12^ vps to 27 patients, adenoviral DNA was detected in blood from day 1 post-infusion to day 28 by qPCR [39]. Another study employing an adenoviral vector containing wild-type p53 (*Ad5CMV-p53*) reported circulating adenovirus at 24 h post infusion and p53 transgene detectable in tumor [37]. Combined, these data suggest systemic administration of replication-defective adenoviral constructs in humans is both safe and potentially effective in targeting human tumors.

The observed specificity of *Ad5H2E-PPE1(3x)* and *Ad5H2E-mVEGFR-ASMase* gene therapy for neo-angiogenic tumor endothelium relative to surrounding normal tissue is the core element of our proposed model for use of this drug in human tumor therapy. We previously reported that normal tissue endothelium constitutes a primary target for SDRT, mediating radiation-induced tissue damage in multiple normal organs, such as lung, brain and GI tract [15], [25], [26]. Thus, sparing normal non-dividing endothelium within radiation-sensitive organs should facilitate use of our ASMase gene therapy, yielding enhanced SDRT tumor cure without increasing normal tissue toxicity.

This paradigm should be viewed in the context of the current state-of-the-art of SDRT. Despite the promise from ongoing clinical trials that image-guided SDRT can ablate >90% of human tumors regardless of tumor type [5], [8], use of SDRT is largely limited by tumor proximity to critical normal organs. For example, a recent study of 204 patients receiving 24 Gy SDRT for spinal metastases abutting the esophagus revealed 15% acute or late esophageal toxicities, including 6.8% severe toxicities (grade 3–4 – i.e. stenosis, ulceration, and trachea-esophageal fistula formation) [40]. Comprehensive analysis of these complications yielded a set of guidelines for spinal SDRT, contraindicating treatment if ≥3.78 cm^3^ of esophagus is anticipated collaterally exposed to 12 Gy, ≥2.5 cm^3^ to 14 Gy, ≥1.87 cm^3^ to 15 Gy, ≥0.11 cm^3^ to 20 Gy, or if any point of esophagus might receive ≥22 Gy. Similar contraindications to SDRT have been developed for other tumor/normal tissue combinations [41]. Radiosensitizing *Ad5H2E-PPE1(3x)-ASMase* or *Ad5H2E-mVEGFR2-ASMase* gene therapy, which does not affect the radiosensitivity of normal tissues, might hypothetically enable SDRT dose de-escalation to levels below the inherent threshold for tumor-adjacent normal tissue toxicity, while still affording curative radiation dosing. A recent Phase I-II trial for extracranial metastases revealed a steep dose-response curve for SDRT, with local cure progressing from 25% to 83% between 18–24 Gy [8], a range of only 6 Gy. This range is similar to the dose shift conferred by *Ad5H2E-PPE1(3x)-ASMase* (**Fig. 6**). If *Ad5H2E-PPE1(3x)-ASMase* radiosensitization can convert 24 Gy tumor cure to a 15–18 Gy iso-effect, it would significantly reduce or eliminate risk of collateral normal tissue toxicity, as this dose level is at or below the threshold of toxicity for most critical human organs [42]. Such dose reduction should significantly increase the number of patients amenable to SDRT for current indications, but more importantly might facilitate recruitment of new indications, currently ineligible due to risk of severe toxicity. Such indications might include treatment of primary prostate cancer that frequently adjoining the rectum and/or urethra, primary and metastatic lung cancer adjacent to or engulfing mediastinal structures (i.e. bronchi, esophagus, peripheral nerves, blood vessels, pericardium), and abdominal tumors in the proximity of or adhering to intestinal loops. Additionally, pre-operative ASMase gene therapy plus low-dose SDRT to shrink tumor may decrease surgical morbidity or increase surgical options for tumors considered inoperable because of proximity to nearby normal structures. As such, the gene therapy agents developed here, or similar constructs, may have significant clinical benefit, providing practical and safe tumor-specific radiosensitization. Further, the magnitude of SDRT sensitization observed with ASMase gene therapy appears superior to other experimental and clinical radiation sensitizers in development [43]–[46], indicating high promise for use in clinical studies.

## Materials and Methods

### Ethics statement

The Institutional Animal Care and Use Committee of Memorial Sloan-Kettering Cancer Center (IACUC protocol 92–10–038) approved this study. All procedures performed comply with provisions of the Animal Welfare Act. Memorial Sloan-Kettering Cancer Center's animal care and use program is administered by the Research Animal Resource Center (RARC). The program has been fully accredited by the Association of Assessment and Accreditation of Animal Care, International (AAALAC) since 1967, is registered with the USDA, and has an approved assurance on file with the Office of Laboratory Animal Welfare, NIH (OLAW).

### Cell culture

HUVEC and HCAEC (Cambrex; East Rutherford, NJ) were cultured in EBM-2 medium supplemented with EGM-2 or EGM-2 MV SingleQuot supplement, respectively (Cambrex, East Rutherford, NJ) at 37°C in a humidified 5% CO_2_ chamber. HeLa cells obtained from ATCC (Rockville, MD), were cultured in DMEM supplemented with 10% fetal bovine serum (FBS), 100 U/ml penicillin, 100 µg/ml streptomycin, and 2 mM L-glutamine at 37°C in a humidified 5% CO_2_ chamber. Cloned populations of BAEC, obtained as described [47] were cultured in DMEM supplemented with 5% normal calf serum, 100 U/ml penicillin, 100 µg/ml streptomycin, and 2 mM L-glutamine at 37°C in a humidified 5% CO_2_ chamber. Irradiation of cultured cells was carried out in Shepherd Mark I irradiator containing a ^137^Cs source at a rate of 2.08 Gy/min. For experiments involving examination of events occurring under 10 min, irradiation was carried out closer to the ^137^Cs source at a rate of 13.1 Gy/min. Where indicated, cells were pre-incubated with 1 ng/ml basic FGF (R&D Systems, Minneapolis MN). In each study, an aliquot of cells was stained with trypan blue to assess viability.

### Apoptosis quantitation

Apoptosis was assessed *in vitro* by examining morphologic changes in nuclear chromatin. Stimulated cells were fixed with 2% paraformaldehyde, washed with phosphate buffered saline (PBS), and stained with 100 μl of 24 μg/ml *bis*-benzimide trihydrochloride solution (Hoechst #33258; Sigma-Aldrich, Milwaukee WI) for 10 min. Morphologic changes of nuclear apoptosis including chromatin condensation, segmentation and compaction along the periphery of the nucleus, or appearance of apoptotic bodies were quantified using an Axiovert S-100 Zeiss fluorescence microscope as per [48].

Apoptosis was quantified *in vivo* in endothelium of tumor specimens following TUNEL staining as described [12]. Several different endothelial markers were evaluated for use on 5 µm paraffin-embedded sections in combination with TUNEL; the best signal to noise ratio was achieved with a monoclonal antibody against the endothelial cell surface marker MECA-32 (Developmental Studies Hybridoma Bank, developed under the auspices of the NICHD and maintained by The University of Iowa).

### Detection of ceramide-rich platforms (CRPs)

Platforms were detected as described [49]. Tumor endothelial cells, following elution from the MACS separation column, were washed with PBS and re-suspended in 0.3×10^6^ cells/ml of DMEM supplemented with 0.2% human albumin. Following irradiation, cells were incubated at 37°C for indicated times and fixed with 2% paraformaldehyde for 15 min at 4°C. Prior to staining, non-specific sites were blocked by incubation in PBS containing 2% FBS for 20 min. Following a PBS wash, cells were stained for surface ceramide or surface ASMase, using anti-ceramide antibody MID 15B4 IgM (1∶50 dilution, Alexis Biochemicals) or polyclonal rabbit anti-ASMase antibody 1598 [49] (1∶100 dilution) respectively, for 1 h at 4°C. Irrelevant mouse IgM or rabbit IgG were used as isotype controls. Following three washes with PBS containing 0.05% Tween-20, cells were stained for platform detection with Texas Red-conjugated anti-mouse IgM or Cy3-conjugated anti-rabbit IgG (1∶300 dilution, Roche Molecular Biochemicals), respectively, for 1 h at 4°C. Lastly, cells were washed 3× in PBS containing 0.05% Tween-20 and mounted in fluorescent mounting medium (Dako, Carpenteria CA). Fluorescence was detected using an Axiovert S-100 Zeiss fluorescence microscope equipped with a SPOT digital camera. Percent of cells containing platforms, i.e. those in which the fluorescence condenses onto less than 25% of the cell surface, was determined by counting 150–250 cells per point.

### Ceramide quantitation

Irradiated BAEC were incubated at 37°C and terminated by placing cells on ice. Subsequently, cells were washed 2× with cold PBS, and lipids extracted by addition of scraped cells in methanol to an equal volume of chloroform and 0.6 volume of buffered saline /EDTA solution (135 mM NaCl, 4.5 mM KCl, 1.5 mM CaCl_2_, 0.5 mM MgCl_2_, 5.6 mM glucose, 10 mM HEPES pH 7.2, 10 mM EDTA). Ceramide was quantified by the diacylglycerol kinase assay as per [50].

### ASMase activity

ASMase activity was quantified in BAEC by radioenzymatic assay using [^14^C-methylcholine]sphingomyelin (Amersham Biosciences, Piscataway, NJ) as substrate, as described with minor modifications [51]. Briefly, following stimulation, cells were placed on ice. Conditioned media containing secreted proteins collected over 18 h was filtered through 40 μm filter mesh (BD Falcon), and concentrated 10 fold using Amicon Ultracel-30 (Millipore, Billerica, CA) concentrator (molecular weight cut off – 30,000). Cells were washed with ice cold PBS and lysed in PBS containing 0.2% Triton X-100. For assaying activity, post nuclear supernatants or conditioned media were incubated with [^14^C-methylcholine]sphingomyelin substrate (0.026 μCi/9.5 nmol) in 250 mM sodium acetate, pH 5.0 supplemented with 0.1% Triton X-100 and 1 mM EDTA or 0.1 mM ZnCl. Reactions were terminated after 1 h with CHCl_3_: MeOH∶1N HCl (100∶100∶1, v/v/v), and product was quantified by a Beckman Packard 2200 CA Tricarb scintillation counter.

### Preparation of recombinant replication-deficient adenoviruses

For *H2E-PPE1(3x)-GFP* or *H2E-PPE1(3x)-ASMase,* the murine *preproendothelin-1 3x [PPE1(3x)]* promoter (VBL Therapeutics, Israel [21]) was inserted into a multiple cloning site cassette produced by us in a modified shuttle vector *pEGFP-1* (Clonetech) in BamHI-NotI restriction sites. Subsequently, H2E enhancer (see Supporting Materials and Methods in File S1 for details) was ligated into the HindIII restriction site of the shuttle vector upstream of the *PPE1(3x)* promoter, generating the GFP expression vector. Human *ASMase* gene, originating from *PCMV1*, kindly provided by Genzyme (Cambridge, MA), was inserted into the *Not-I* site downstream of the enhancer-promoter sequence. Lastly, the *H2E-PPE1(3x)-ASMase* or *H2E-PPE1(3x)-GFP* cassette was subcloned into the Mlu-1 restriction site within the multiple cloning site of *pVQAs-NpA* vector, provided by ViraQuest, Inc (North Liberty, IA). Replication-deficient recombinant adenoviruses (serotype 5, E1/E3 deleted) were prepared by ViraQuest Inc. using the RAPAd.I system as described [52]. Viral stocks were stored at −80°C at concentration of 10^9^–10^11^ PFU/ml. Adenoviruses used as empty vector control, *Ad5Empty*, or non-tissue-specific control *Ad5CMV(GFP)* were purchased from ViraQuest Inc.

*H2E-mVEGFR2-GFP-IE* and *H2E-mVEGFR2-ASMase-IE* expression vectors were generated in our laboratory. A synthetic gene was engineered by Integrated DNA Technologies, Inc. This gene contained a KpnI restriction site, the *H2E* enhancer, the murine *VEGFR2* promoter, and the 5′end of the human *ASMase* gene through a naturally occurring BlpI restriction site. This sequence was synthesized for seamless transition from the 5′ UTR of murine *VEGFR2* promotor through the first human *ASMase* codon. The existing *H2E-PPE1(3x)-ASMase* in the *pVQAs-NpA* vector was modified by insertion of a 511 bp intronic enhancer (IE) element from the first intron of the murine *VEGFR2* gene into the BglII site downstream of the poly A signal. Thereafter, the *VEGFR2*-*ASMase* fusion was inserted replacing *H2E*, *PPE(3x)* and the 5′end of *ASMase*. A second synthetic gene was similarly engineered to contain a KpnI restriction site, *H2E*, murine *VEGFR2* promotor, and full-length *GFP* ending in a SpeI restriction site. This cassette was inserted into the modified *pVQAs-NpA* vector at the KpnI and SpeI sites. Replication-deficient recombinant adenoviruses termed *Ad5H2E-mVEGFR2-GFP-IE* or *Ad5H2E-mVEGFR2-ASMase-IE* were prepared and handled as above.

### Adenovirus infection in vitro

BAEC (10^5^ cells/well), HUVEC, HCAEC and HeLa cells (0.7×10^5^ cells/well) were plated in 12-well tissue culture treated plates 24 h before infection. Prior to plating, cells were resuspended in their respective culture media, as indicated above, supplemented with 10% NCS (BAEC) or 10% FBS (HUVEC, HCAEC and HeLa). Infections were performed by incubation with 1, 5 and 10 multiplicity of infection (MOI) of *Ad5Empty* or *H2E-PPE1(3x)-GFP* in a total volume of 400 μl of culture media supplemented with 2% NCS (BAEC) or 2% FBS (HUVEC, HCAEC and HeLa). After 12 h, virus containing media was removed and cells were incubated with culture media supplemented with 5% NCS (BAEC) or 10% FBS (HUVEC, HCAEC and HeLa) in a total volume of 1 ml. To measure GFP reporter expression, at the indicated times, cells were detached by a 2 min incubation in 0.05% Trypsin (Cambrex, East Rutherford, NJ), resuspended in PBS supplemented with 0.5% FBS and GFP expression was assessed by flow cytometric analysis. 7-AAD Viability Dye (BD Biosciences) was used to quantify dead cells. 20,000 cells were analyzed on a FACScan flow cytometer (BD Biosciences) with CellQuest software (Becton Dickinson). To measure impact of gene therapy on radiation response, at 5 days after *Ad5Empty* or *Ad5H2E-PPE1(3x)-ASMase* infection, BAEC were switched to DMEM containing 0.2% human albumin and after 18 h irradiated as described above.

### Mice and in vivo experiments

sv129/BL6^SKI^ *asmase^−/−^* mice were inbred in our colony and genotyped using a modification of the protocol described by Horinouchi *et al.* [53], employing a revised PA2 primer (5′-GGCTACCCGTGATATTGC-3′), and 35 cycles of PCR amplification, each at 94°C for 15 sec, 64°C for 30 sec, and 68°C for 90 sec. Wild-type, sv129/BL6^JAX^ male mice, 6–8 weeks old, were purchased from Jackson Laboratories (Bar Harbor, ME). Mice were housed at the animal core facility of Memorial Sloan-Kettering Cancer Center. This facility is approved by the American Association for Accreditation of Laboratory Animal Care and is maintained in accordance with the regulations and standards of the United States Department of Agriculture and the Department of Health and Human Services, National Institutes of Health.

MCA/129 fibrosarcoma and B16 melanoma cells were maintained in DMEM high glucose supplemented with 10% FCS, 100 U of penicillin/ml and 100 μg streptomycin/ml in 10% CO_2_ at 37°C. Cells (10^6^) were resuspended in PBS and injected subcutaneously into the right flank as described [12]. Once tumors reached the size 80–100 mm^3^, 1×10^10^ PFU of *Ad5Empty*, *H2E-PPE1(3x)-GFP*, *Ad5CMV-GFP* or *H2E-PPE1(3x)-ASMase* was administered intravenously by a single tail vein injection. For irradiation experiments, four or five days post virus administration, an indicated dose of IR was delivered using a Philips MG-324 X-ray unit at 118.3 cGy/min (50 cm source to skin distance). Mice were lightly sedated with ketamine (0.1 mg/g) and xylazine (0.02 mg/g) and only tumor, surrounding skin and subcutaneous tissues, were exposed, the rest of the mouse was shielded using a specialized lead jig. Tumor volume, based on caliper measurements, was calculated daily according to the formula of Kim *et al.* [54]. Tumor cure is defined as no detectable tumor confirmed at autopsy.

### Endothelial cell isolation

Tumor endothelial cells were isolated following a modification of a technique published by Garcia-Barros *et al.* [12]. MCA/129 fibrosarcoma tumors were dissected from the hind limbs, washed twice in PBS, cut into small pieces and incubated in cocktail containing 2 mg/ml collagenase A (Roche), 250 μg/ml elastase (Roche) and 25 μg/ml DNAseI (Roche) in DMEM supplemented with 1% FCS, 20 mM HEPES (pH 7.4), and Penicillin (100 U/ml)-Streptomycin (100 μg/ml) at 37°C with gentle shaking. After 45 min, the tumor digest was filtered sequentially through 100, 70 and 40 μm nylon filter mesh (BD Falcon). Filtered samples were washed twice with 0.5% BSA in PBS and centrifuged at 800×g for 5 min three times at 4°C. Cells were separated based on density through a preformed 30% Percoll gradient (Amersham Pharmacia Biotech) at 800×g for 30 min at 4°C. This step removes platelets and RBCs, which can cause clumping of the magnetic beads. Cells at the top of the gradient were removed carefully, and washed twice with 0.5% BSA in PBS. For negative selection to remove hematopoietic cells, MACS microbeads (Miltenyi Biotec Inc.), conjugated to antibody directed against hematopoietic cell surface marker CD45 were incubated with the fraction obtained from the Percoll gradient for 15 min at 4°C at a dilution 1∶10, as indicated by the manufacturer. The total Percoll gradient fraction-antibody-conjugated MACS microbeads incubation was applied to the MACS LS Separation columns (Miltenyi Biotec Inc.), and the column was washed with 9 ml of 0.5% BSA in PBS. This process was repeated using the flow through to increase specific binding. Flow cytometric analysis showed that 90% of the cells retained on the column were positive for CD45. Thereafter, the fluent fraction was incubated with MACS microbeads conjugated to anti-mouse CD146 antibody (LSEC microbeads; Miltenyi Biotech) for positive selection of tumor endothelial cells (1∶10 dilution). Following a 15 min incubation at 4°C, cells were washed and applied to the MACS LS Separation columns, as described above. Tumor cells pass through the column, while endothelial cells remain bound to the beads. Thereafter, the column was detached from the magnet, and the endothelial cells bound to microbeads were eluted in 0.5% BSA in PBS and subsequently passed through the column to increase specific binding. Analysis by flow cytometry showed that the final eluate from the magnetic column contained 80% pure endothelial cells based on the binding of endothelial specific markers VEGFR2, CD31 (BD Biosciences) and VE-cadherin (clone Bv13, kindly provided by ImClone).

### Tissue GFP expression quantification

Tissues were dissected five days after *in vivo* infection with *AdH2E-PPE1(3x)-GFP* or *Ad5H2E-mVEGFR2-ASMase*, washed with PBS and fixed in freshly prepared 4% paraformaldehyde in PBS at 4°C overnight. Following paraffin embedding, 5 μm thick sections were obtained by microtomy, adhered to polylysine-treated slides and deparaffinized by heating at 90°C for 10 min and at 60°C for 5 min, followed by two xylene washes for 5 min. Automated immunostaining (Discovery XT automated machines) of the tissue sections was performed using 10 μg/ml of a rabbit polyclonal anti-GFP antibody (Molecular Probes). To quantify GFP expression in endothelium, GFP-stained tissues were subsequently stained with 3 μg/ml of monoclonal antibody against the endothelial cell surface marker MECA-32. Fluorescence was detected using an Axiovert S-100 Zeiss fluorescence microscope equipped with a SPOT digital camera. For quantifying GFP positive endothelial cells, immunostained slides were scanned using the Mirax scanner and generated images were analyzed using the Mirax viewer software (Carl Zeiss, Inc.)

**Crypt microcolony survival assay** was performed according to Withers and Elkind [27].

### Statistics

Values are expressed as mean ± SD unless otherwise noted. Paired, two-tailed students t tests were calculated using Prism v4.

## Supporting Information

Figure S1 Overexpression of human ASMase in BAEC primarily increases the activity of Zn^2+^-dependent S-ASMase. Cellular homogenates and serum-free conditioned media were harvested from BAEC infected with *Ad5Empty* or *Ad5H2E-PPE1(3x)-ASMase* and assayed for ASMase activity at pH 5.0 using [^14^C-methylcholine]sphingomyelin as a substrate in the presence of 1 mM EDTA or 0.1 mM Zn^2+^. Data (mean ± SEM) are collated from 3 independent experiments performed in triplicate. (TIF)

Figure S2 Optimization of *Ad5H2E-PPE1(3x)-ASMase* administration. 1×10^10^ PFU of *Ad5H2E-PPE1(3x)-GFP* was administered intravenously to MCA/129 fibrosarcoma- (A–C) and B16 melanoma- (B) bearing mice and tumors were excised at 2–5 days (A) or at 5 days (B,C) post viral administration. Reporter gene expression was assessed following immunostaining of tumor sections with anti-GFP and Meca-32, as described in Materials and Methods. Data (mean ± SEM) represent GFP-positive endothelial cells collated from 20 fields/tumor and 2–4 tumors/group. (TIF)

Figure S3 Figure S3. Infection with *Ad5H2E-mVEGFR2-GFP* induces GFP expression specifically in endothelial cells. Endothelial cells (BAEC, HUVEC and HCAEC) were infected with *Ad5H2E-mVEGFR2-GFP.* GFP expression was measured in live cells following detachment 24, 48 and 72 h post-infection by flow cytometry. Of note, Hela and Jurkat cells express GFP minimally ≤8% at all times up to 72 h. (TIF)

Figure S4 Intravenous administration of *Ad5H2E-mVEGFR2-GFP* results in GFP expression selectively in tumor endothelium. 2×10^10^ PFU of *Ad5Empty* (control), *Ad5H2E-mVEGFR2-GFP* or *Ad5CMV-GFP* were administered i.v. to MCA/129 fibrosarcoma-bearing sv129/BL6 mice. Five days post viral administration, normal tissues (A) and tumor tissue (B) were excised and GFP expression was visualized by standard fluorescence microscopy following staining with anti-GFP (green; A, B) and anti-MECA-32 (red; B) antibodies, as described in Materials and Methods. Shown are representative 20× images of 20 fields analyzed per sample. Note background autofluorescence in the kidney specimens. (TIF)

Figure S5 Overexpression of ASMase in tumor endothelium using the murine VEGFR2 promoter radiosensitizes MCA/129 fibrosarcoma to IR. 2×10^10^ PFU of *Ad5H2E-mVEGFR2-ASMase* was administered i.v. to MCA/129 fibrosarcoma-bearing sv129/BL6^JAX^ *asmase^+/+^*mice. Five days post virus administration tumors were locally irradiated with 33 Gy. Response of MCA/129 fibrosarcoma to treatment with *Ad5H2E-mVEGFR2-ASMase* and IR (black lines) or IR alone (gray lines) is presented as tumor volume. N equals number of animals per group. Tumors were measured daily up to 40 days and twice weekly thereafter. Tumor cure was confirmed by local biopsy. (TIF)

File S1 Supporting Materials and Methods, Supporting Results, and Supporting References. (DOCX)
