# Supplementary figures and images for: Adenoviral Transduction of Human Acid Sphingomyelinase into Neo-Angiogenic Endothelium Radiosensitizes Tumor Cure
Source: PLoS One. 2013 Aug 2;8(8):e69025. doi: 10.1371/journal.pone.0069025 (PMC3732255; doi:10.1371/journal.pone.0069025)

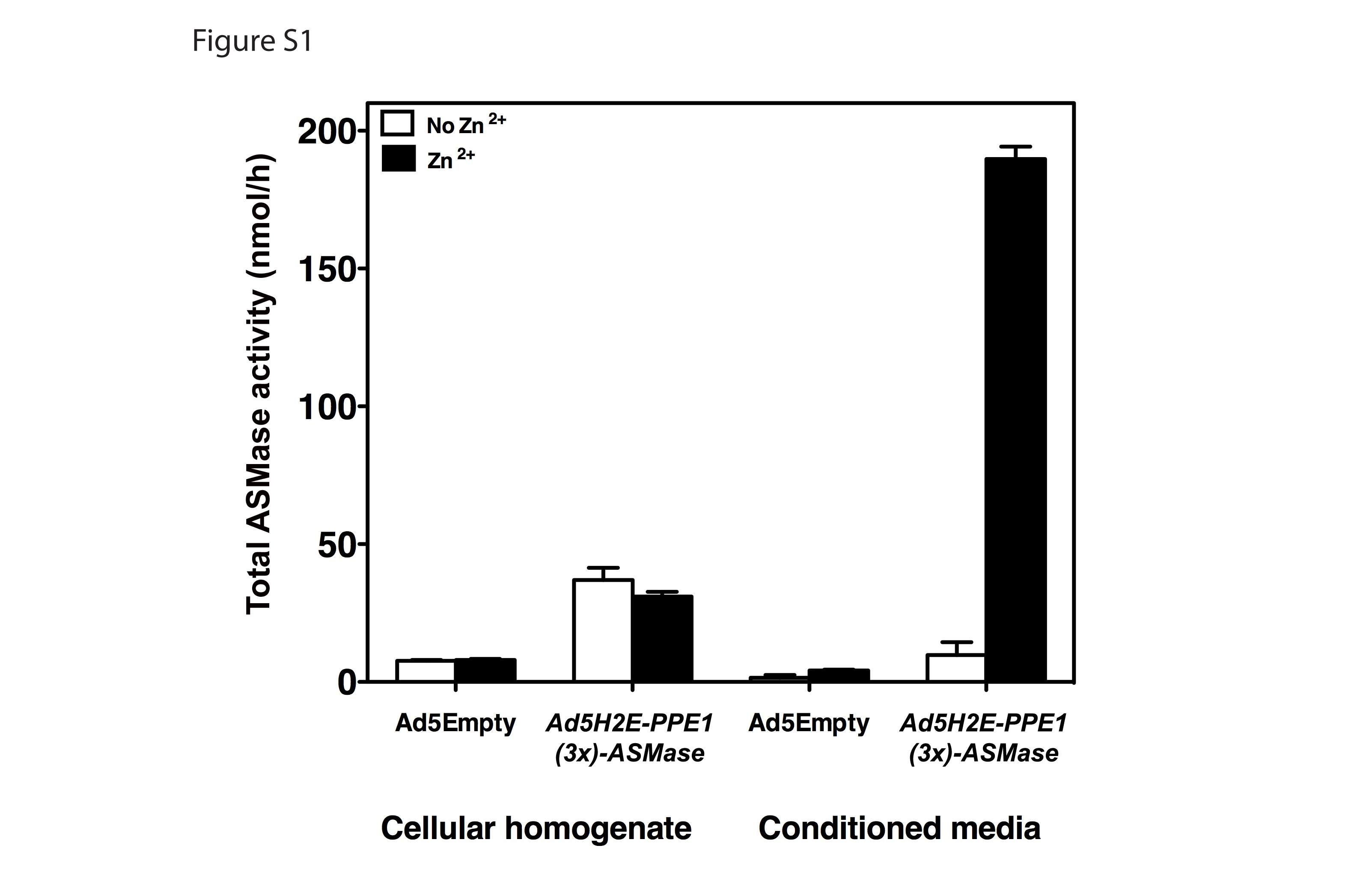

Supplement: Figure S1 — (TIF) [file pone.0069025.s001.tif]

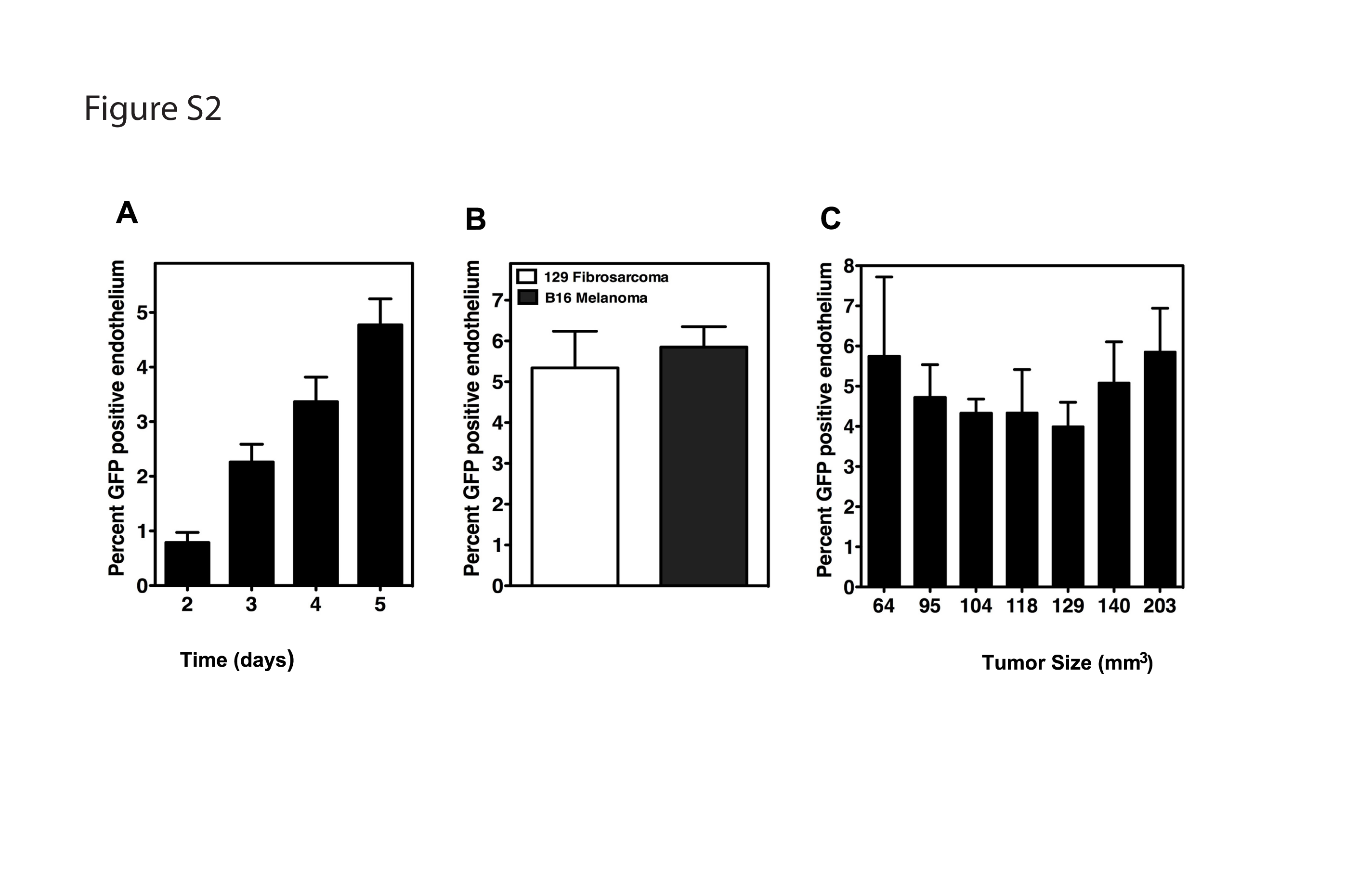

Supplement: Figure S2 — (TIF) [file pone.0069025.s002.tif]

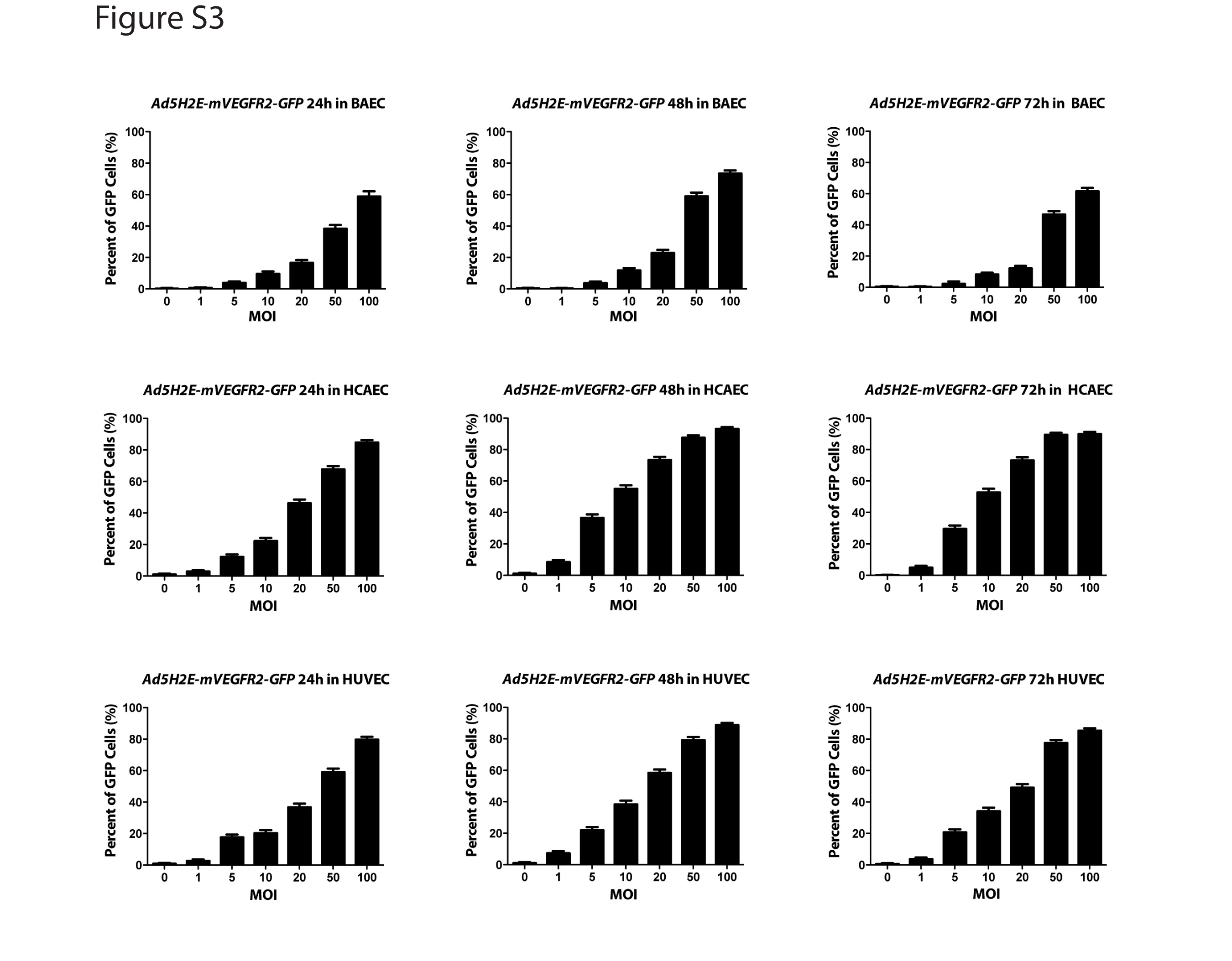

Supplement: Figure S3 — (TIF) [file pone.0069025.s003.tif]

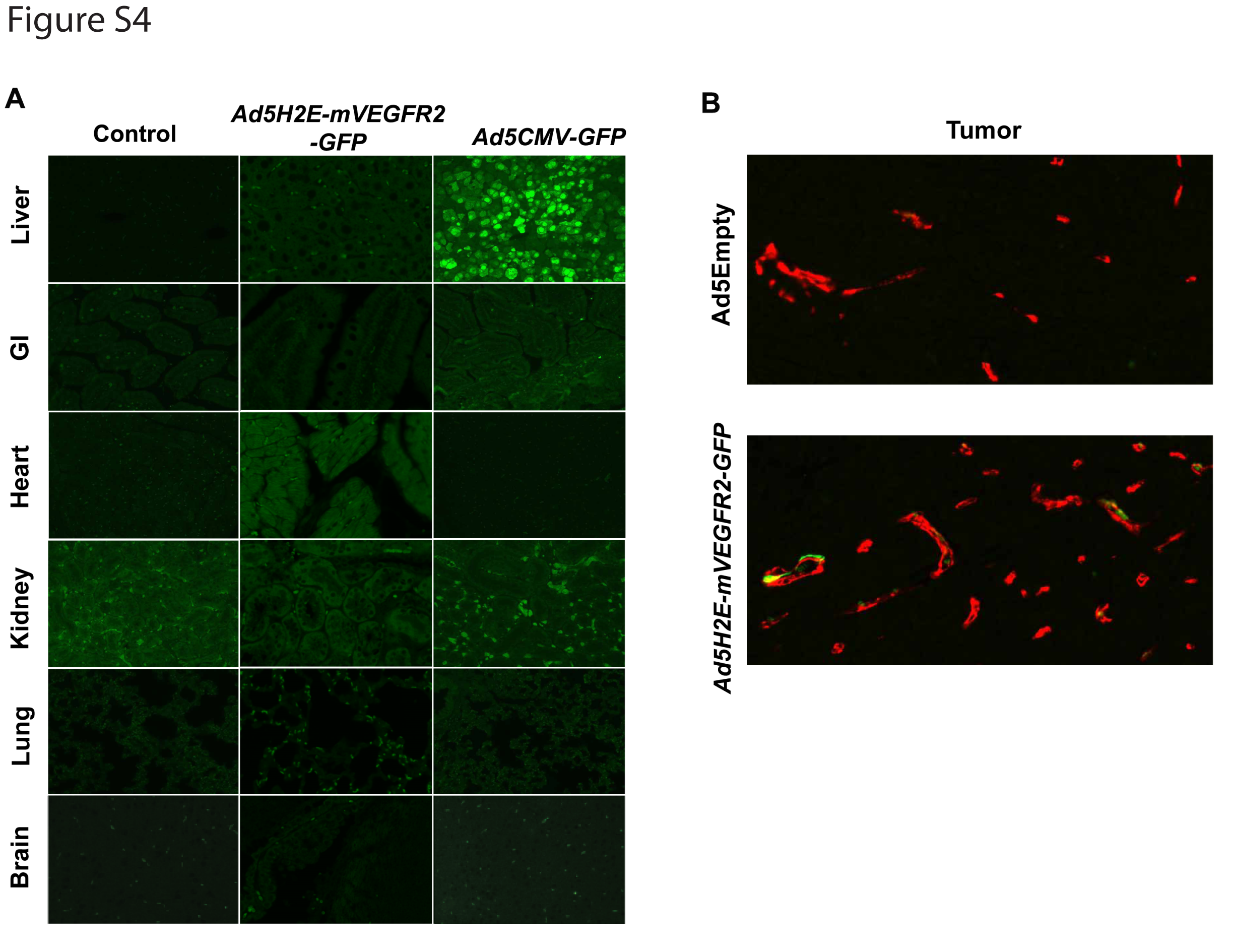

Supplement: Figure S4 — (TIF) [file pone.0069025.s004.tif]

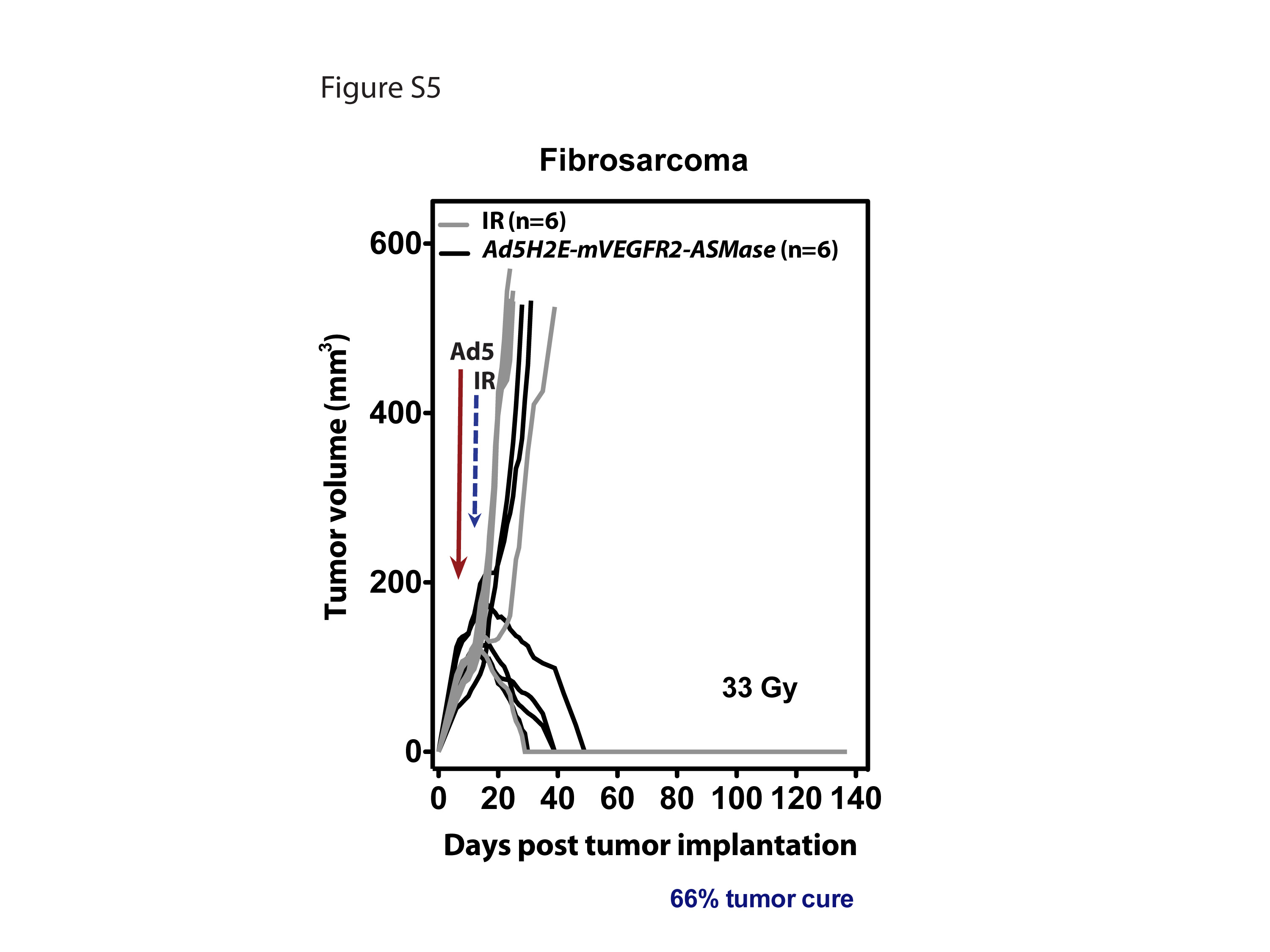

Supplement: Figure S5 — (TIF) [file pone.0069025.s005.tif]
