# Supplementary material for: Adenoviral Transduction of Human Acid Sphingomyelinase into Neo-Angiogenic Endothelium Radiosensitizes Tumor Cure
Source: PLoS One. 2013 Aug 2;8(8):e69025. doi: 10.1371/journal.pone.0069025 (PMC3732255; doi:10.1371/journal.pone.0069025)
Supplement: File S1 — (DOCX) [file pone.0069025.s006.docx]

Supporting Materials and Methods

Preparation of the *H2E* Enhancer: The synthetic *HIF-2α-Ets-*1 DNA binding element was constructed by producing a fusion fragment composed of the *HIF2α* and *ETS1* motifs originally located in the murine *VEGFR2* (Flk-1) endothelial specific promoter [[1](#_ENREF_1),[2](#_ENREF_2)]. Two complementary 132 bp fragments synthesized by GeneLink Ltd. were annealed using Klenow fragment (NEB) according to manufacturer’s instructions. Double strand DNA fragments were purified by elution from a 3% agarose gel using Qiagene kit. The *HIF-2α-Ets-1* DNA binding element (termed *H2E*) was inserted upstream of *PPE1(3x)* or *mVEGFR2* promoters.

**Preparation of the Murine *VEGFR2* *(Flk-1)* Promoter Construct:** The murine promoter was cloned from healthy C57Bl6 mouse liver DNA using two primers flanking the promoter region: -623 (5'agttcacaaccgaaatgt3') and from +457 (5'agatctcgagatgctcaat3') [[3](#_ENREF_3)], which resulted in 1080bp DNA fragment length. The promoter was sub-cloned into the modified *pEGFP-1* using BaHI and NotI restriction sites, following by subcloning the *GFP-Luciferase* (*GFPLuc*) fusion reporter gene into NotI site.

**In Vitro Plasmid Transfection:** BAEC were cultured for 24-48 h prior to 70% confluence and transfected with plasmids containing *GFP* or *GFPLuc* downstream of a specific endothelial promoter (*wtPPE-1-Luciferase, PPE1(3X)-GFPLuc, H2E-PPE1(3X)-GFPLuc, FLK-1-GFP-Luc,* or *H2E-FLK-1-GFPLuc*) using Lipofectamine 2000 (Invitrogen) according to manufacturer’s instructions. Forty-eight hours post transfection, luciferase activity was measured by luminometer.

**Supporting Results**

**The *H2E* enhancer increases target gene expression controlled by *PPE1(3x)* under normoxic and hypoxic conditions**

Luciferase expression under control of various different promoters described in Supporting Materials and Methods showed that the modified pre-proendothelin promoter, *PPE1(3x)*, yields higher Luciferase activity/mg protein than either of the *VEGFR2* promoters *Flk-1* or *KDR* (data not shown). Moreover, insertion of the *H2E* enhancer upstream of *PPE1(3x)* increased luciferase activity 3.3-fold under normoxic conditions and 1.32-fold additionally under hypoxic conditions, following transient transfection of *PPE1(3x)-GFPLuc* and *H2E-PPE1(3x)-GFPLuc*, respectively, into BAEC. We attribute enhanced expression in normoxic conditions to the presence of the Ets-1 binding site, as the Ets-1 transcription factor is expressed in endothelium, induced by bFGF and VEGF [[4](#_ENREF_4)]. An increase in target gene expression in hypoxic conditions is likely attributable to the presence of the *HIF-2α* binding element. Based on these data, we selected the *H2E-PPE1(3x)-ASMase* construct as the preferred candidate for initial adenovirus development.

**Supporting Figure Legends**

**Fig. S1. Overexpression of human ASMase in BAEC primarily increases the activity of Zn^2+^-dependent S-ASMase.** Cellular homogenates and serum-free conditioned media were harvested from BAEC infected with *Ad5Empty* or *Ad5H2E-PPE1(3x)-ASMase* and assayed for ASMase activity at pH 5.0 using [^14^C-methylcholine]sphingomyelin as a substrate in the presence of 1 mM EDTA or 0.1 mM Zn^2+^. Data (mean ± SEM) are collated from 3 independent experiments performed in triplicate.

**Fig. S2. Optimization of *Ad5H2E-PPE1(3x)-ASMase* administration.** 1x10^10^ PFU of *Ad5H2E-PPE1(3x)-GFP* was administered intravenously to MCA/129 fibrosarcoma- (**A**-**C**) and B16 melanoma- (**B**) bearing mice and tumors were excised at 2-5 days (**A**) or at 5 days (**B**,**C**) post viral administration. Reporter gene expression was assessed following immunostaining of tumor sections with anti-GFP and Meca-32, as described in Materials and Methods. Data (mean ± SEM) represent GFP-positive endothelial cells collated from 20 fields/tumor and 2-4 tumors/group.

**Fig. S3. Infection with *Ad5H2E-mVEGFR2-GFP* induces GFP expression specifically in endothelial cells.** Endothelial cells (BAEC, HUVEC and HCAEC) were infected with *Ad5H2E-mVEGFR2-GFP.* GFP expression was measured in live cells following detachment 24, 48 and 72 h post-infection by flow cytometry. Of note, Hela and Jurkat cells express GFP minimally ≤8% at all times up to 72 h.

**Fig. S4. Intravenous administration of *Ad5H2E-mVEGFR2-GFP* results in GFP expression selectively in tumor endothelium.** 2x10^10^ PFU of *Ad5Empty* (control), *Ad5H2E-mVEGFR2-GFP* or *Ad5CMV-GFP* were administered i.v. to MCA/129 fibrosarcoma-bearing sv129/BL6 mice. Five days post viral administration, normal tissues (**A**) and tumor tissue (**B**) were excised and GFP expression was visualized by standard fluorescence microscopy following staining with anti-GFP (green; **A**, **B**) and anti-MECA-32 (red; **B**) antibodies, as described in Materials and Methods. Shown are representative 20x images of 20 fields analyzed per sample. Note background autofluorescence in the kidney specimens.

**Fig. S5. Overexpression of ASMase in tumor endothelium using the murine VEGFR2 promoter radiosensitizes MCA/129 fibrosarcoma to IR.** 2x10^10^ PFU of *Ad5H2E-mVEGFR2-ASMase* was administered i.v. to MCA/129 fibrosarcoma-bearing sv129/BL6^JAX^ *asmase^+/+^*mice. Five days post virus administration tumors were locally irradiated with 33 Gy. Response of MCA/129 fibrosarcoma to treatment with *Ad5H2E-mVEGFR2-ASMase* and IR (black lines) or IR alone (gray lines) is presented as tumor volume. N equals number of animals per group. Tumors were measured daily up to 40 days and twice weekly thereafter. Tumor cure was confirmed by local biopsy.

**Supporting References**

1. Kappel A, Schlaeger TM, Flamme I, Orkin SH, Risau W, et al. (2000) Role of SCL/Tal-1, GATA, and ets transcription factor binding sites for the regulation of flk-1 expression during murine vascular development. Blood 96: 3078-3085.

2. Elvert G, Kappel A, Heidenreich R, Englmeier U, Lanz S, et al. (2003) Cooperative interaction of hypoxia-inducible factor-2alpha (HIF-2alpha ) and Ets-1 in the transcriptional activation of vascular endothelial growth factor receptor-2 (Flk-1). J Biol Chem 278: 7520-7530.

3. Rönicke V, Risau W, Breier G (1996) Characterization of the endothelium-specific murine vascular endothelial growth factor receptor-2 (Flk-1) promoter. Circ Res 79: 277-285.

4. Sato Y, Teruyama K, Nakano T, Oda N, Abe M, et al. (2001) Role of transcription factors in angiogenesis: Ets-1 promotes angiogenesis as well as endothelial apoptosis. Ann N Y Acad Sci 947: 117-123.
